# The Kings of Spain

**Published:** 1894-10-13

**Authors:** 


### The Psychologist in History.

### IV.—THE KINGS OF SPAIN.

In almost every case of lunacy there is a history behind of defective intellect, but so few can tell much of their ancestors that this is often difficult to trace. Only in the most exalted stations are family histories so kept as to bear true testimony to the growth, development, and variations of hereditary characteristics. Such a history we have of the kings of Spain —a history which tells how continued intermarriage brought about the degeneration and extinction of a noble race, in spite of occasional outbursts of brilliancy which made Spain and the Holy Roman Empire for the nonce the greatest forces in Western Europe.

In the fourteenth century there were in the Peninsula three kings named Pedro, each of whom was surnamed "the Cruel. From the intermarriage of their descendants we get the royal line which ruled Spain when Spain was at its greatest. John II. of Castile married Isabella of Portugal, and their daughter, Isabella of Castile—the Queen Isabella of Christopher Columbus—married Ferdinand of Aragon. John II. was of weak intellect himself, and his wife was insane for years before she died. Yet in Isabella of Castile the neurotic tendency was arrested, and to her faith in a genius beyond the common we owe the great New World. Ferdinand, her husband, was cold and shrewd; he was a monarch, he desired to be an autocrat, and by the suppression of the free institutions of Spain, added to his own and his wife's piety, he succeeded in forming that Spain which drove out the industrious Jews and the intelligent Moors, and became the happy hunting-ground of the Inquisition.

Ferdinand and Isabella were succeeded by a daughter, Juana. This lady is said to have become mad with grief at the death of her husband. Jealous of him in life, she was jealous of him after he was dead, and hung over his corpse, keeping all other women away. There had been symptoms of mental instability before this, but from this time forth she was a prey to the most depressing and degraded forms of madness, degenerating into habits so disgusting that in that age, had they been indulged in by any other than a queen, they would have been punished by the application of the lash.

Yet this was the mother of the Emperor Charles V. That Charles V. possessed genius is undeniable. Amid the conflicting elements of the Renaissance, the enmity and jealousy of ambitious rivals, he maintained and increased the power of the empire. But he was subject to epilepsy, and he was not only unscrupulous —no uncommon thing among the politicians of his time—but superstitious, and subject to melancholia. These characteristics seem to have had much to do with his abdication and retirement to a monastery when in the height of his power. The intellectual powers of Charles V. were transmitted to his illegitimate children, Don John of Austria, and Margaret, the austere but vigorous Regent of the Netherlands. But Charles married his cousin, Isabella of Portugal, granddaughter of Ferdinand and Isabella, and the hereditary neurosis, thus intensified, showed itself clearly in their children.

The Emperor's son and successor, Philip II., was not indeed insane, but he was delicate physically, and of a morose, obstinate, and superstitious character. He also married his cousin, Mary of Portugal, a descendant of his own insane grandmother, Juana. These two were the parents of Don Carlos. The unfortunate fate of this prince has cast a halo of romance around him, and the poets who have told his history have pictured something much superior to the man as he lived. Don Carlos was, in person, short of stature halting in his gait, and low-browed; he had a slight hump and articulated with difficulty. He was cruel, delighting to roast alive animals taken in the chase. No tutor could make him other than illiterate, and he failed completely in those manly exercises which in princes are held in more esteem. His father regarded him as insane, but when the insanity showed itself in threats to take his life, he imprisoned him. The prince then tried more than once to commit suicide; he failed, but the over-indulgence in eating which hastened his end might pass for something akin.

Don Carlos never came to the throne, and it was his father's fourth wife, who was also his niece, who was the mother of the next king, Philip III. He and his successor, Philip IV., were much alike in character. Both were lazy and weak, and left the government of a kingdom which then held all the possibilities of the Western world to the hands of capriciously chosen favourites. Yet they were better than the son and successor of Philip IV., Charles II., of whom Macaulay says that he thoroughly enjoyed only two things, "a horse with its bowels gored out and a Jew writhing in the flames." So dull of intellect was he that he did not know the names of the provinces and cities of his kingdom. He was twice married, but had no children, and bequeathed his throne to his grand-nephew, Philippe de Bourbon, a bequest which plunged all Europe into the sanguinary war of the Spanish succession. The Spanish Bourbons, like their predecessors, showed insanity and imbecility, and under them the great power of Spain dwindled and disappeared.

The taint in the blood of the Spanish kings was undeniable, but it was aggravated by frequent intermarriages, and by that strict etiquette which isolated the king in the midst of his subjects, and kept him from that equal intercourse with his fellows which keeps man's views of life balanced and sane. No solitary life is ever healthy, whether it is the solitude of the king on his throne or of Stylites on his pillar.

---

A substitute for carbolic acid has recently been suggested by Dr. J. W. Charteris, of Glasgow, who claims many advantages for Trecresol (or Trikersol).

Certain freaks of fashion are now claiming a defence on a scientific ground. It is entertaining to hear from Chicago that the red parasol—all the rage in that city—is an efficient freckle-preventer. "The actinic rays of the sun," as the American Press has pointed out, and which are the real cause of this pigmentation, "are intercepted in passing through the red medium!"

